# Direct Growth of Bacteria in Headspace Vials Allows for Screening of Volatiles by Gas Chromatography Mass Spectrometry

**DOI:** 10.3389/fmicb.2018.00491

**Published:** 2018-03-20

**Authors:** Collin M. Timm, Evan P. Lloyd, Amanda Egan, Ray Mariner, David Karig

**Affiliations:** Applied Physics Laboratory, Johns Hopkins University, Laurel, MD, United States

**Keywords:** VOC, GC-MS, *Pseudomonas aeruginosa*, microbial diversity, skin microbiome

## Abstract

Bacterially produced volatile organic compounds (VOCs) can modify growth patterns of eukaryotic hosts and competing/cohabiting microbes. These compounds have been implicated in skin disorders and attraction of biting pests. Current methods to detect and characterize VOCs from microbial cultures can be laborious and low-throughput, making it difficult to understand the behavior of microbial populations. In this work we present an efficient method employing gas chromatography/mass spectrometry with autosampling to characterize VOC profiles from solid-phase bacterial cultures. We compare this method to complementary plate-based assays and measure the effects of growth media and incubation temperature on the VOC profiles from a well-studied *Pseudomonas aeruginosa* PAO1 system. We observe that *P. aeruginosa* produces longer chain VOCs, such as 2-undecanone and 2-undecanol in higher amounts at 37°C than 30°C. We demonstrate the throughput of this method by studying VOC profiles from a representative collection of skin bacterial isolates under three parallel growth conditions. We observe differential production of various aldehydes and ketones depending on bacterial strain. This generalizable method will support screening of bacterial populations in a variety of research areas.

## Introduction

Volatile organic compounds (VOCs) can act as long distance signaling molecules between microbes and other organisms and have been shown to impact attraction of biting insects to mammals (Mboera et al., 1997), recruitment of herbivores, and pollinating insects to plants (Pichersky and Gershenzon, 2002), and growth of rival microbes in a community (Lazazzara et al., 2017; Rybakova et al., 2017). For example, bacterial metabolism of 2-butanediol and acetoin can modify root growth patterns in plants (Ryu et al., 2003) while VOCs from plants can, in turn, inhibit decay by microorganisms (Utama et al., 2002). Similarly, in the human gut, short chain fatty acids (SFCAs) produced by commensal and pathogenic bacteria contribute to the development and progression of ulcerative colitis, asthma, and a range of other diseases (Machiels et al., 2014; Arrieta et al., 2015; Ríos-Covián et al., 2016). Notably, bacteria are not the only microbes to participate in VOC signaling. Indeed, fungi have been shown to display quorum sensing behavior by the production of tyrosol (Chen et al., 2004) and other alcohols (Hogan, 2006). The importance of VOCs in microbial systems, coupled with current interest in microbiome research, requires methods for rapid characterization of volatile compounds from microbial isolates. In this work, we describe a method to enable higher throughput discovery and analysis of microbial volatiles by gas chromatography mass spectrometry (GC-MS).

One important facet of VOC research is the development of novel diagnostic markers for human disease. Analysis of bacterially produced VOCs, for example, has been applied successfully for detection of *Pseudomonas aeruginosa* infections in the lungs of cystic fibrosis (CF) patients. In particular, when paired with *in vitro* culture techniques (Labows et al., 1980; Scott-Thomas et al., 2010; Gilchrist et al., 2012a), increased levels of hydrogen cyanide, 2-aminoacetophenone, 2-nonanone, and 2-undecanone in the breath of CF patients corresponds to infection with *P. aeruginosa* (Labows et al., 1980; Zechman and Labows, 1985; Enderby et al., 2009; Savelev et al., 2010; Gilchrist et al., 2011). Volatile markers have also been suggested for *S. aureus* (Filipiak et al., 2012), *H. pylori* (Pavlou et al., 2000), *Plasmodium* infection (Trowell et al., 2015), and *B. cepacia* (Gilchrist et al., 2012a,b). Meanwhile, *in vitro* characterization of produced volatiles has been performed for pathogenic strains of, *S. pneumoniae, Escherichia faecalis, Klebsiella pneumoniae*, and *E. coli*, all of which may enable early detection of infections (Bos et al., 2013).

Another important aspect of VOC research is signaling to biting arthropods. Humans release a variety of volatile compounds that vary with age (Gallagher et al., 2008) and emission site (Amann et al., 2014) and that contribute to attraction of mosquitos, flies, and ticks (Hammack et al., 1987; Mboera et al., 1997; Braks et al., 1999). Biting arthropods, in turn, can be vectors for a large number of diseases, including malaria (Cowman et al., 2016), dengue (Simmons et al., 2012), sleeping sickness (Kennedy, 2013; Brun et al., 2017), Lyme disease (Petnicki-Ocwieja and Brissette, 2015), and hemorrhagic fevers (Ergönül, 2006). Characterization of human VOC profiles shows that production of lactic acid, 2-methylbutanoic acid, tetradecanoic acid, 4-hydroxy-3-methoxybenzoic acid, a variety of *N,N* amines, hexanedioic acid, oxazole, 2-methylisothiazole, and octanal is associated with being more attractive to mosquitoes while production of limonene, 2-phenylethanol, methylpentanol, methyliodide and 2-ethyl-1-hexanol is associated with being less attractive to mosquitoes (Bernier et al., 2002; Verhulst et al., 2013). Notably, microbes that comprise the human skin microbiome are major producers of many of the VOCs that induce differential attraction to mosquitoes (Verhulst et al., 2011a,b). Thus, an understanding of microbial contribution to VOCs from the human skin may enable active control of attractiveness to biting insects through microbiome modulation.

Current methods to study VOCs can provide detailed identification of multiple compounds from complex mixtures with unknown components. GC-MS, for example, enables separation of compounds and is ideal method for novel compound discovery. Unfortunately, existing technologies tend to be limited in terms of sample throughput. This impacts their usefulness for characterizing VOCs under ranges of conditions or under dynamic conditions, for example during microbial growth. Existing protocols for studying VOCs involve either direct sampling of the atmosphere from fermentation vessels (Wu et al., 2016) or else indirect sampling via solvent extraction or adsorption of VOCs to glass beads (Bernier et al., 2002), solid phase micro extraction (SPME) fibers (Gallagher et al., 2008), or polydimethylsiloxane (PDMS) membranes (Riazanskaia et al., 2008). Either of these techniques can be applied to monocultures or co-cultures, with the latter including interaction studies in which organisms share an atmosphere (Ryu et al., 2003; Lazazzara et al., 2017; Rybakova et al., 2017). Collection methods are typically followed by thermal desorption and injection of VOCs into GC/MS systems for chemical analysis. SPME, in particular, is becoming increasingly common. This is in part because SPME collection can be performed without physically contacting the sample and in part because a range of portable SPME fibers have dramatically simplified field use. However, SPME as a sampling technique is highly dependent on vapor concentrations. Samples with low concentrations of volatile compounds require long SPME incubation times, hindering use for some applications. VOC sampling rates can be further increased by the use of proton transfer reaction mass spectrometry (PTR-MS), in which the separation step of analysis is omitted, greatly reducing sample processing time (Romano et al., 2015). This approach enables near real-time monitoring of sample VOCs with greater quantification accuracy than GC-MS and has been employed in the monitoring levels of anesthetics in breath during surgery (Critchley et al., 2004), soil VOC exchange rates (Asensio et al., 2007) and decomposition of plant litter (Gray et al., 2010; Ramirez et al., 2010), the production of volatiles by microbial processes for food production and food safety (Holm et al., 2013; Blasioli et al., 2014; Makhoul et al., 2015; Capozzi et al., 2016), and has been shown to be parallelizable for up to four distinct microbial cultures (Bunge et al., 2008). Further, PRT-MS is an ideal choice for monitoring biological production processes including fuel production from the fungi *Ascocoryne sacroides* (Mallette et al., 2012), VOC production from the plant pathogen *Xanthomonas campestris* (Weise et al., 2012), response of *Mycobacterium smegmatis* cultures to antimicrobial agents (Crespo et al., 2011), growth of *Thalassiosira pseudonana* (Kameyama et al., 2011), and differential production of VOCs from *Staphylococcus aureus* under multiple nutrient conditions (O'Hara and Mayhew, 2009). PTR-MS and GC-MS techniques have been used in tandem with GC-MS being used to identify compounds, and PTR-MS used for monitoring and quantification (Weise et al., 2012; Blasioli et al., 2014). These techniques have also been used in parallel to compare methods directly in ability to detect and quantify microbial produced VOCs (Mallette et al., 2012; Holm et al., 2013; Blasioli et al., 2014). While PTR-MS can provide real-time analysis of microbial volatiles, we recognized the need for higher throughput analysis using GC-MS for discovery based mass spectrometry and comparative analysis of unknown compounds across multiple microbial cultures.

A common approach to study microbial diversity is the screening for a variety of phenotypes. In this work we present a high throughput method to characterize VOC profiles from solid-phase bacterial cultures. The method involves culturing bacterial samples directly in headspace vials that can be handled by an autosampler and then sampling the headspace using SPME. Our method allows for long-term incubation of bacterial culture and includes trapping of VOCs, increasing the ability to detect compounds produced at low concentrations. In this work we measure how SPME fiber film composition affects VOC detection from bacterial cultures, and we then compare our method to common plate sampling techniques. Increased throughput of sample analysis allows us to demonstrate our method by studying VOC production under different temperature incubations, and using different growth media. We conclude by screening VOC profiles from human skin microbiome representatives growing under diverse growth conditions. This generalizable method will support screening of bacterial populations in a variety of research areas.

## Methods

### Bacterial strains and culture conditions

Bacterial strains used in this study are listed in Table 1 and were maintained on tryptic soy agar (BD, Franklin Lakes, NJ, USA) and grown in tryptic soy broth (BD, Franklin Lakes, NJ, USA) at 30°C. MOPS minimal media (#M2101, #M2101, #G0520), MOPS minimal media plus EZ supplement (#M2104, #M2101, #M2101, #G0520), or MOPS rich media (#M2103, #M2104, #M2101, #M2101, #G0520), were used according to manufacturer's instructions (Teknova, Hollister, CA, USA), with 1.5% molecular biology grade agar (Teknova, Hollister, CA, USA). Where described, 1 g/L of yeast extract (Sigma-Aldrich, St. Louis, MO, USA) was added to MOPS minimal media and dissolved prior to autoclaving. Detailed media formulations are included in Table S1. The headspace vials used in this study were 75.5 × 22.5 mm, 20 mL, screw neck, clear glass vials (Gerstel Inc., Linthicum, MD, USA, product number #093640-036-00) with magnetic screw caps with blue silicone/PTFE septa (Gerstel Inc., Linthicum, MD, USA, product number #093640-040-00). For growth in headspace vials, autoclaved vials were filled with 8 mL prepared culture media which was allowed to solidify at room temperature. Prepared vials were stored at 4°C for no longer than 2 weeks prior to use. Vials were inoculated with ~100 CFU in 50 μL PBS. Inoculated vials were incubated at either 30 or 37°C and were placed onto the autosampling tray for automated incubation of the SPME fiber.

**Table 1:** Bacterial strains and ordering information.

**Species**	**Strain**	**Source**	**Ordering Id**.
*Pseudomonas aeruginosa*	PAO1-LAC	ATCC	ATCC 47085
*Staphylococcus epidermidis*	FDA strain PCI 1200	ATCC	ATCC 12228
*Staphylococcus epidermidis*	Fussel	ATCC	ATCC 14990
*Staphylococcus epidermidis*	SK135	BEI	HM-118
*Rhodococcus erythropolis*	SK121	BEI	HM-116
*Acinetobacter radioresistans*	SK82	BEI	HM-107
*Brevibacterium epidermidis*	NCDO 2286	ATCC	ATCC 35514
*Corynebacterium xerosis*		ATCC	ATCC 373

### Gas chromatography and mass spectrometry

The SPME fiber extractions and chromatographic data were acquired using a Gerstel MPS robotic autosampler system (Gerstel Inc., Linthicum, MD, USA) with a Thermo Trace 1310 GC and a Thermo TSQ 8000 triple quadrapole mass spectrometer (GC/MS) using Xcalibur software (Thermo Fisher, Waltham, MA, USA). The analytical column used was a Restek Rtx-5 fused silica column (15 m × 0.25 mm × 0.25 μm) (Restek Corporation, Bellefonte, PA, USA). The SPME fibers evaluated for extracting bacterial VOCs were purchased from Supelco (Supelco Inc., Bellefonte, PA, USA) and consisted of a 7 μm PDMS fiber (Fiber A, green), a 100 μm PDMS fiber (Fiber B, red), a 50/30 μm DVB–CAR–PDMS fiber (Fiber C, gray), and a 65 μm PDMS-DVB fiber (Fiber D, pink). The fibers were loaded into an autosampler syringe held within the Gerstel MPS robotic sampler arm and were conditioned before use within the Gerstel syringe heating block, as directed by manufacturer's guidelines. To perform automatic sampling, a preparation method was created within the Gerstel software, Maestro QQQ. This method programs the robotic autosampler to transfer 20-mL headspace vials containing media and bacterial cultures into a 30°C heated incubator for a brief sample incubation. SPME fiber extraction occurs while the headspace vial is warmed within the 30°C incubator. In our experiments, the fiber was exposed to the headspace for 45 min prior to desorption into the GC/MS inlet. Thermal desorption was performed in a 250°C PTV inlet, with a Siltek Metal Liner (2 mm ID × 2.75 OD × 120 mm Length). The PTV inlet was operated in splitless mode for 1.20 min, followed by a 50 mL/min split. The GC carrier gas for the analytical system was Helium, operating in Constant Flow mode with a flow rate of 1.50 mL/min. The GC oven parameters used were a 40°C initial oven temperature with a 3 min hold, ramping at 8°C/min to 120°C with no hold, and then a final ramp of 35°C/min to 260°C with a 3 min hold. The MS parameters used for detection were an MS transfer line temperature of 250°C and an ion source temperature of 200°C. The MS was operated in full scan with a scan range of 50–400 amu at a rate of 5 scans/s. Following fiber desorption in the GC inlet, the autosampler arm was moved to the syringe heating block for a final SPME fiber bake-out at 200°C for 15 min, to ensure the fiber was clean post-injection.

### Peak identification and quantification

Both Thermo Xcaliber software (version 2.2, SP1.48, 2011) and the National Institute of Standards and Technology's (NIST 2005) Automated Mass Spectral Deconvolution & Identification System (AMDIS version 2.62) were used for peak identification. AMDIS allows for automated, unbiased matching of m/z spectra against a custom library, whereas Thermo Xcaliber qualitative analysis allows for manual peak picking and library matching. AMDIS search parameters included a minimum search factor of 80%, using a high threshold from 50 to 400 m/z. A component width of 12, adjacent peak subtraction of 2, high resolution and very high sensitivity were defined for deconvolution parameters. The custom search library (Supplemental Data) was initially constructed using 351 known skin VOCs as described in the literature (Bernier et al., 2000; Haze et al., 2001; Gallagher et al., 2008; Jiang et al., 2013; Tait et al., 2014). For peaks that AMDIS did not find a match to in our custom library, Xcaliber qualitative analysis was used to find a match against the full NIST library (version 2.0 d, 2005). Once identified with at least 80% confidence, the match was added to our custom library. In total, 461 compounds comprised our final VOC library, and all analyses were performed using AMDIS a second time against the final VOC library (Table S1). Reported hits against our final custom library were reported with >80% confidence. High abundance peaks from *P. aeruginosa* PAO1 cultures were confirmed by comparison to chemical standards including co-injection analysis. For signal to noise analysis, peaks were identified using Xcaliber Genesis peak detection, with valley detection enabled and a minimum peak height (s/n = 2.0), S/N threshold = 0.5 and a minimum percent of highest peak = 1.0. Baseline noise tolerance % was set to 10.0, the minimum number of scans in baseline to 16, and the baseline noise rejection factor to 2.0. The peak S/N cutoff was set to 200, the Rise Percentage was set to 10.0, the Valley S/N was set to 1.0, the Background Recomputation was set to min: 5.0), and the number of scans in background was set to 5.

## Results

### Bacterial growth in headspace vials increases sensitivity

**Figure 1:**
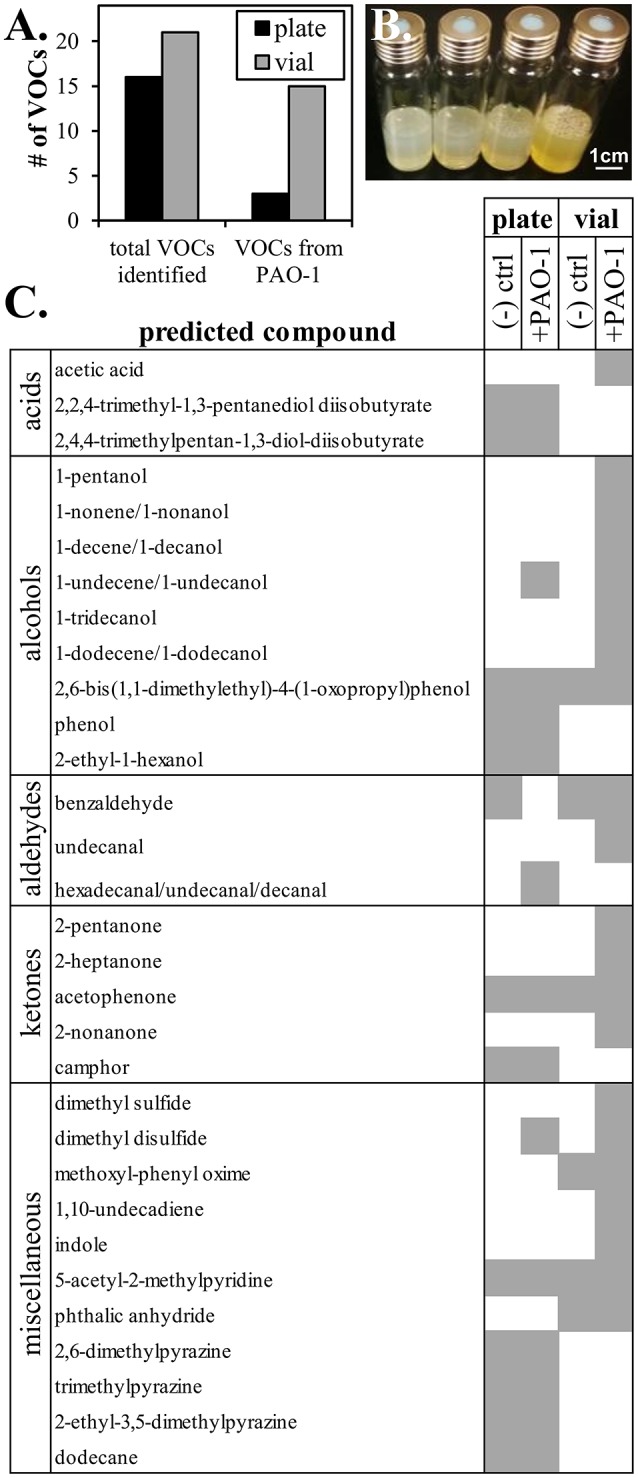
Comparison of current methods with plate based assay for VOC detection. **(A)** Count of peaks identified by plate (traditional) or vial (current) sampling method for VOC detection. **(B)** Headspace vials with agar media and growth of PAO1. Scale bar represents 1 cm. **(C)** Summary of compounds putatively identified from peaks from plate and vial sampling.

Our method consists of generating semisolid growth surfaces in sterilized headspace vials, followed by inoculation with bacteria and incubation at growth temperatures, which are then sampled with a SPME fiber, and autosampled by GC/MS. When compared to standard methods for VOC analysis of bacterial cultures, our headspace approach greatly reduced sampling times. Whereas, 4 h incubation is used for sampling from culture plates (our data), our method enabled SPME collection of VOCs in 40 min. Moreover, our method resulted in volatile profiles that had an increased total number of peaks detected and an increased number of peaks unique to samples incubated with *P. aeruginosa* PAO1 (Figure 1A). Both our methods and the standard approach detected 2-undecanol and dimethyl disulfide in cultures but not in uninoculated controls, consistent with previous studies of *P. aeruginosa* that have identified these compounds as bacterial products. In addition, we detected 1-pentanol, 1-tridecanol, and 2-undecanone as bacterial products using vial sampling (Figures 1B,C) which were found previously in headspace sampling (Labows et al., 1980; Filipiak et al., 2012). We also observed that a peak identified as benzaldehyde had a significantly decreased peak intensity in samples incubated with *P. aeruginosa* PAO1 compared to uninoculated controls, consistent with bacterial metabolism of this substrate. To determine increase in sensitivity, we calculated signal to noise ratios for major peaks identified in *P. aeruginosa* PAO1 growth on plates and in headspace vials. For headspace vial sampling, the signal to noise was recorded as 1832 for undecene, 1135 for nonanone, 473 for undecanone, and 338 for undecanol. In the comparative trace for SPME sampling of PAO1 grown on plates using the manual SPME holder held over a petri dish with PAO1, the signal to noise ratio was calculated as 236 for a broad combined undecene/nonanone peak and 170 for the combined undecanone/undecanol peak. Peaks identified from plate sampling were not as cleanly separated further supporting improvement of direct growth in headspace vials.

### SPME fiber composition affects VOC detection

**Figure 2:**
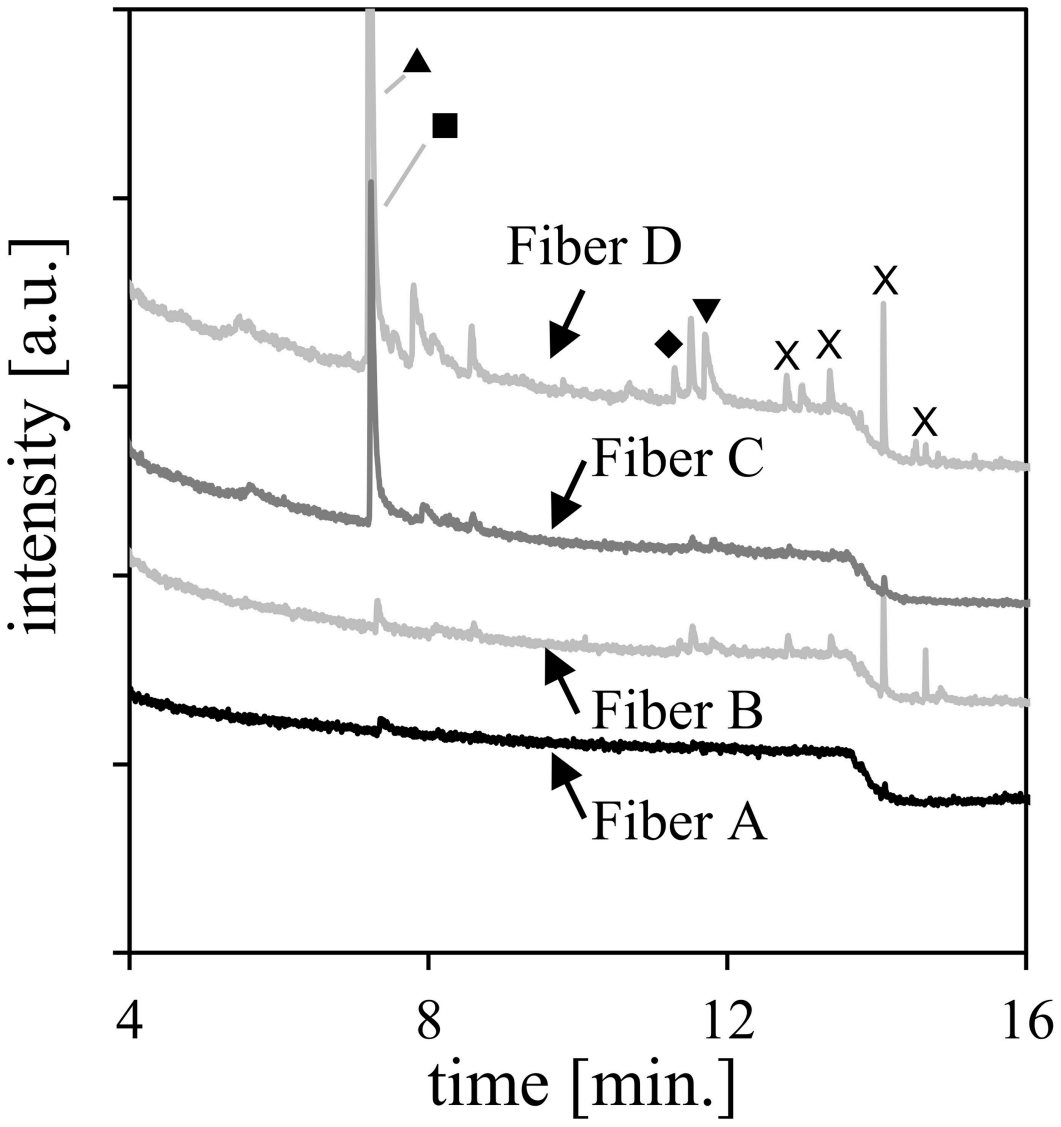
GC/MS chromatograms of *P. aeruginosa* PAO1 cultures extracted with different SPME fiber types at 30°C for 24 h. Peaks from *P. aeruginosa* PAO1 are designated by symbols: ▴ = 1-undecene; ■ = 2-nonanone; ♦ = 2-undecanone; ▾ = 2-undecanol. Peaks labeled as X are identified as siloxane contaminants.

To test how SPME fiber composition affects VOC detection from bacterial samples, individual samples of a 24 h culture of *P. aeruginosa* PAO1 were tested against four SPME fiber types. All fibers used contained polydimethylsiloxane (PDMS) as a base component. Fiber A was comprised of a thin layer of PDMS (7 μm), while Fiber B had a thicker layer of PDMS (100 μm). Fiber C included a divinylbenzene layer (50 μm) and a carboxen layer (30 μm). Finally, Fiber D included only a divinylbenzene layer (65 μm). We observed the largest number of peaks using Fiber D and the smallest number using Fiber A (Figure 2). However, Fiber C was able to extract the long chain VOCs associated with *P. aeruginosa* nearly as well as Fiber D. Fiber C was chosen for the remaining experiments because the inclusion of carboxen layer was thought to provide a wider range of chemical capture.

### Incubation temperature alters volatile profiles and timing

**Figure 3:**
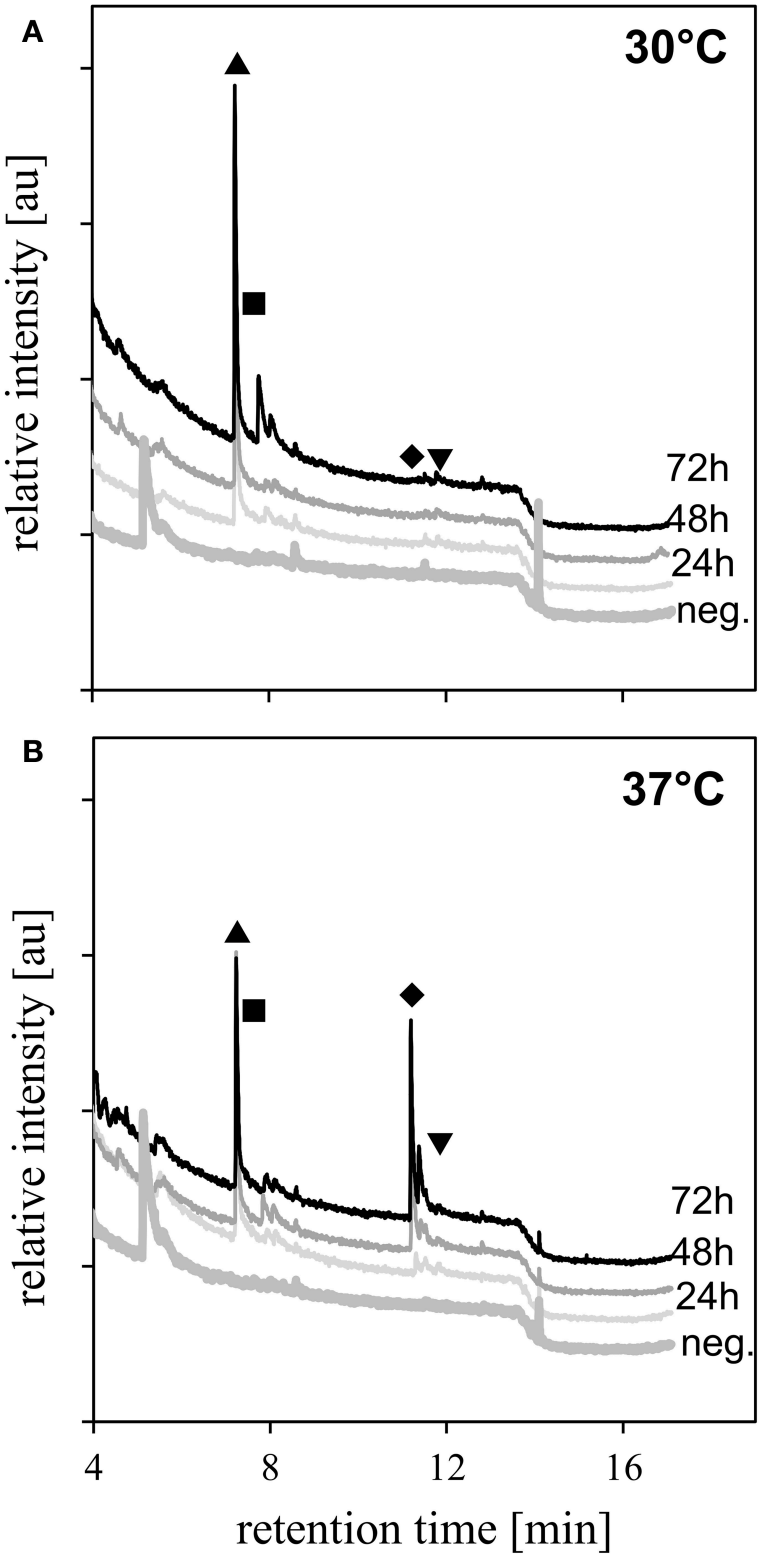
Volatiles from *P. aeruginosa* PA01. **(A)** 30°C incubation, **(B)** 37°C incubation. Negative controls (labeled as “neg.”) were uninoculated and measured daily, (day 1 negative control only shown) for clarity. Peak identifications confirmed with co-elution of chemical standards are designated by symbols: ▴ = 1-undecene; ■ = 2-nonanone; ♦ = 2-undecanone; ▾ = 2-undecanol.

To determine effects of growth temperature on VOC production, inoculated vials were incubated at either 30 or 37°C. Further, because temperature is known to impact microbial growth dynamics, time-course data was collected. To obtain time-course data, VOC profiles were measured daily by SPME incubation followed by GC/MS (Figure 3 and Figure S1). We confirmed the identifications for the most prevalent VOCs by analysis of pure chemical standards using SPME sampling of headspace vials (Table 2 and Figure S1). At 30°C we observed a peak at 7.2 min. that was identified as 1-undecene, as well as a group of corresponding peaks between 7.8 and 8.2 min primarily consisting of 2-nonanone. The alcohol and ketone peaks increased in intensity over time. At 37°C, we observe an additional group of peaks at 11.4 min, which were identified as 2-undecanone and 2-undecanol. Both the 1-undecene and the other methyl ketones have been previously attributed to the *P. aeruginosa* VOC profile in the literature (Labows et al., 1980; Filipiak et al., 2012). In growth series at both temperatures, we observe multiple peaks, including 3-methylbutanal, 4-(1,1-dimethylethyl)-benzenepropanal, and benzaldehyde, which were reduced in intensity relative to uninoculated controls, suggesting that these compounds were media components metabolized by *P. aeruginosa* during growth.

**Table 2:** Chemical standards and retention times.

**Compound**	**Sigma Aldrich SKU**	**Structure**	**Quantity**	**Retention time**
2-pentanone	W284220	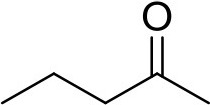	10 μg	1.124 min
1-nonene	74323	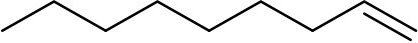	0.1 μg	3.87 min
benzaldehyde	B1334	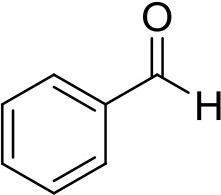	1 ng	5.40 min
1-undecene	242527	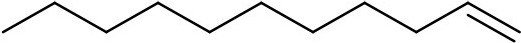	1 ng	8.25 min (Verified by co-injection)
2-nonanone	108731	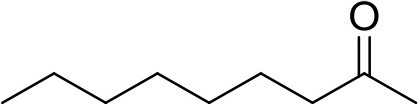	0.1 μg	8.35 min (Verified by co-injection)
2-nonanol	W331503	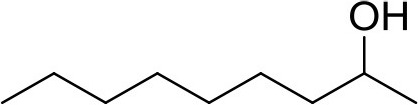	1 ng	9.88 min
1-dodecene	44146	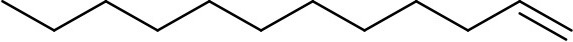	0.1 μg	10.23 min
2-undecanone	U1303	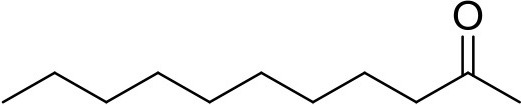	0.1 μg	12.13 min (Verified by co-injection)
undecanal	U2202	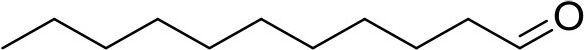	0.1 μg	12.34 min
2-undecanol	8084680100	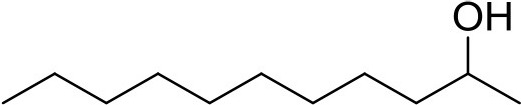	1 ng	13.37 min (Verified by co-injection)

### Media composition affects production of specific VOCs by *P. aeruginosa*

**Figure 4:**
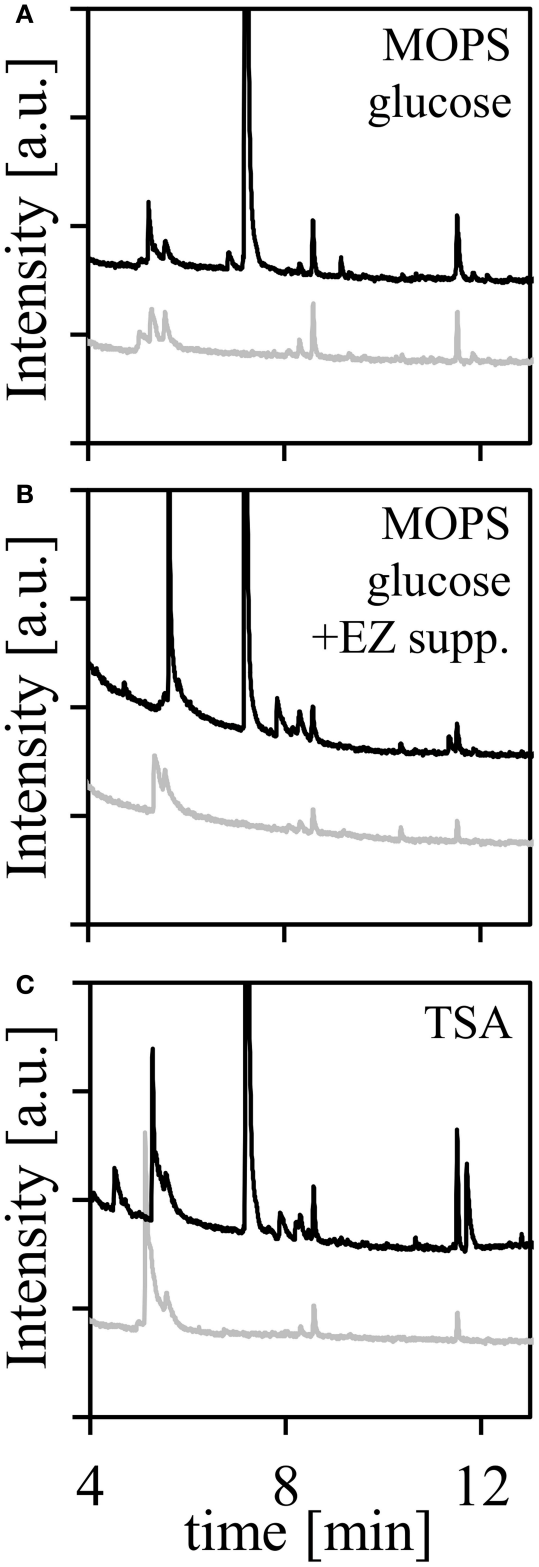
Volatiles from *P. aeruginosa* PAO1 cultures on various media after 1 week incubation. Shown in light gray curves are uninoculated controls, and those in black curves are inoculated with PAO1 and are transposed for separation. **(A)** MOPS minimal media with glucose as the sole carbon source, **(B)** MOPS minimal media with glucose as sole carbon source and EZ supplement (amino acids), **(C)** tryptic soy agar.

We observed multiple peaks which are present in both uninoculated and inoculated samples. We hypothesized that these peaks were inactive components in the media, which led us to investigate the role of media by generating a series of defined media formulations. In addition to defined media for reducing background components, this approach allowed us to study how different environments affect VOC production. The latter may be a result of microbes implementing different metabolic pathways depending on substrate availability. When incubated with *P. aeruginosa* PAO1, we observed the fewest number of background peaks in MOPS Minimal Media using glucose as the sole carbon source (Figure 4). When the EZ supplement (which contains amino acids, nucleotides, and vitamins, Table S1) was added we observed increased production of peaks between 7.8 and 8.1 min, corresponding to 2-nonanone. The rich media (TSA), by contrast, showed increased levels of amino acetophenones. Interestingly, we observed a relatively high abundance of 1-dodecene at 9.2 min in glucose only media, suggesting that components in the EZ supplement may inhibit production of this long-chain alkene.

### Volatiles profiles from diverse representatives from the skin microbiome

**Figure 5:**
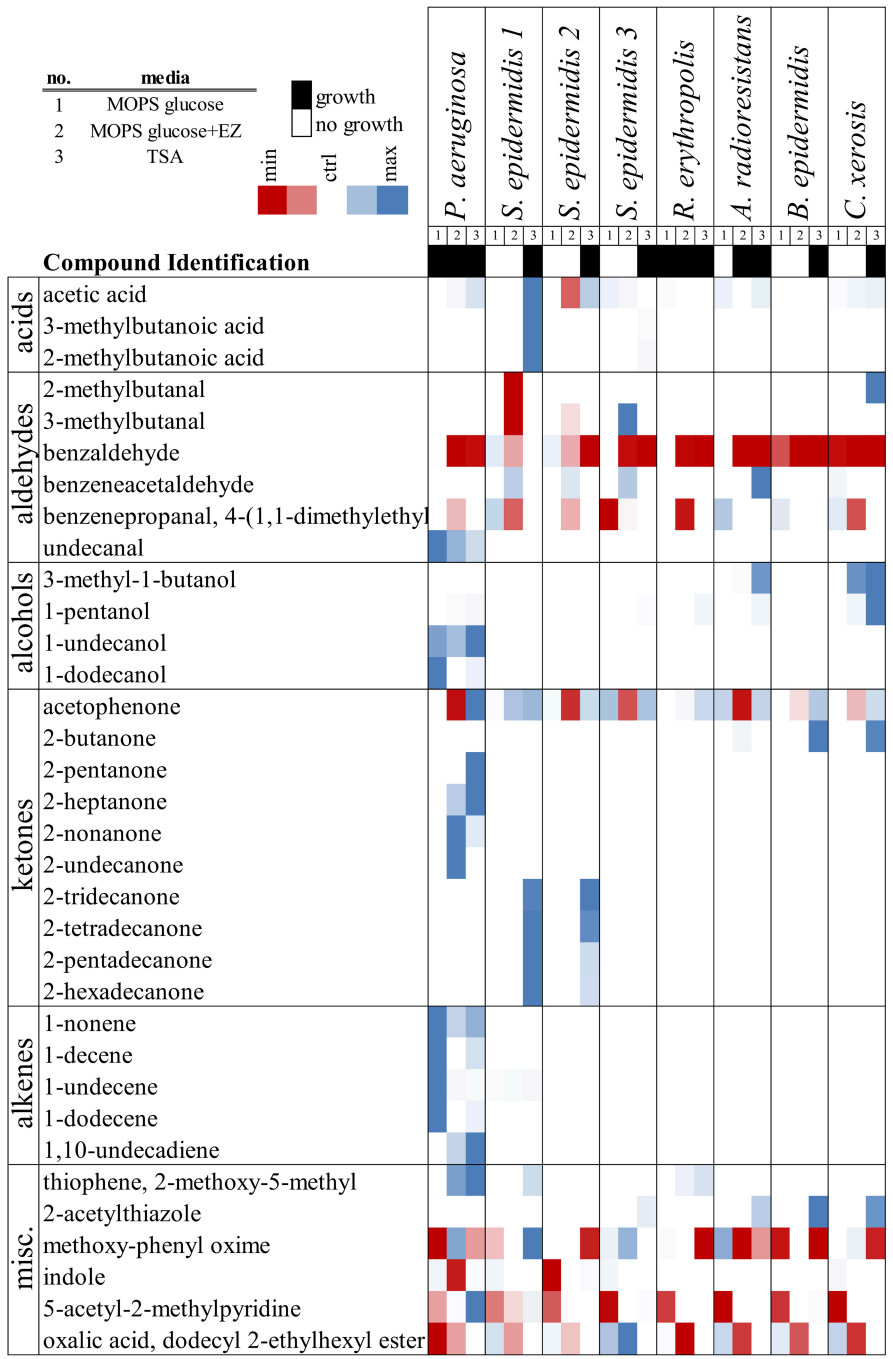
Volatiles from human skin microbiome representatives. Heat map scaled to show relative peak intensities across samples. Growth was determine from visual identification of colonies and is indicated for each media condition. Putative peak identifications are listed and grouped by functional group.

For the measurement of VOC profiles from multiple bacterial strains grown in multiple media types, a more rapid method for characterization was necessary. We selected eight microbial strains representing genera in the human skin microbiome and measured their volatile production after 7 days incubation at 30°C on MOPS glucose, MOPS glucose +EZ, and TSA media formulations (Figure 5). Major VOCs identified from these strains are reported in Figure 4. Consistent hits are only shown if a match in the library was verified both automatically by the AMDIS algorithm as well as by manual confirmation against the NIST library using Thermo Xcaliber software (see Methods). In accordance with the literature, the VOCs observed from bacterial culture were overwhelmingly long-chain fatty acid biosynthesis products. Alkenes, alcohols, ketones, and aldehydes were the majority of all VOCs captured by the SPME GC/MS technique. In general, for samples where no growth was observed, the VOC profiles matched that of the un-inoculated controls.

The utility of our headspace vial analysis of solid culture by SPME was demonstrated by screening multiple bacterial strains grown on multiple media types. The main compound produced by *P. aeruginosa* in all media types at 30°C was 1-undecene, a long-chain alkene. The main VOCs produced by 2 out of 3 strains of *S. epidermidis* were identified as long-chain ketones (TSA media). The first strain of *S. epidermidis* was the only bacteria tested to produce compounds that match the carboxylic acids 3-methylbutanoic acid and 2-methylbutanoic acid. Interestingly, the third strain of *S. epidermidis* did not produce these compounds, but did produce larger than background levels of a compound putatively identified as 3-methylbutanal. Conversely, 2-methylbutanal and 3-methyl butanal were consumed from the media by the first strain of *S. epidermidis* (ATCC 12228). None of the *S. epidermidis* strains had visible growth on MOPS glucose minimal media, and only grew on TSA. *P. aeruginosa*, however, grew on minimal media as well as TSA. In MOPS glucose minimal media, no medium-chain methyl ketones were observed, but medium and long-chain alkenes were produced. In richer media, like TSA and even amino acid-supplemented MOPS glucose media, long-chain alkenes were produced in a lower amount; instead, medium-chain methyl ketones were observed.

The remaining strains studied had more subtle VOC profiles. Despite visible growth in all media types, *R. erythropolis* produced no identifiable VOCs using our methodology. In TSA, *A. radioresistans* produced 3-methyl-1-butanol and benzeneacetaldehyde, but little else. *B. epidermidis* and *C. xerosis* only had visible growth in TSA, and both produced compounds putatively identified as 2-butanone and 2-acetylthiazole. In addition, *C. xerosis* produced 3-methyl-1-butanol and 2-methylbutanal. These four strains did not produce detectable levels of fatty acid biosynthesis products. Fatty acid biosynthesis is necessary for bacterial cell growth to occur, so further reduction of fatty acids to alkenes, alcohols, or methyl ketones may be less common for these strains.

## Discussion

A common approach for studying microbiomes is the culture of representative organisms and screening for phenotypes that may affect interaction with the host or other community members. One mechanism by which microbes interact with other organisms in their environment is through production of VOCs. Current methods for studying bacterially produced VOCs include plate-based growth and manual capturing of VOCs. In this paper, we present an alternative approach based on sampling of headspace vials. Our method provides comparable and often superior results to current methods and reduces required sampling time to <1 h per sample. Using this method, we tested different SPME compositions, collection times and growth conditions to determine how these factors affect VOC detection from *P. aeruginosa* PAO1, a well-studied organism for which VOC detection can be used as a diagnostic for infection. After demonstrating the improved performance of our method and standardizing optimal sampling protocols, we then screened diverse representatives from the human skin microbiome to determine VOC production on different media formulations.

The sampling method that we developed is based on volatile adsorption to SPME fibers. SPME is well suited to microbial VOC analysis because it allows for the sampling of bacterial volatiles from the headspace of solid cultures rather than via liquid injection, either directly from liquid culture or from extracts of agar medium. However, the relatively low concentration of volatiles produced by microbial cultures means that SPME typically requires long incubation times and can be heavily contaminated by VOCs from the environment. As a countermeasure, we have developed a technique that relies on headspace vials. Headspace vials have been employed for identification of volatiles from chemical samples where they have been found effective due to their ability to concentrate VOCs and reduced background contamination benefits provided by the sealed vessel design. Previously, however, headspace vial sampling has only been used for standard chemical profiling. In this paper, we show that a similar approach can be applied to microbial sampling by growing bacterial cultures directly in the headspace vials. When bacteria are grown in headspace vials, buildup of volatiles within the vials enables VOC sampling in <1 h of SPME fiber incubation. By contrast, techniques wherein SPME fibers are held over a culture on a petri dish require incubation for many hours. Notably, we detected major VOCs previously described from *P. aeruginosa* PAO1 using both methods, but observed a larger signal to noise ratio and fewer contaminating VOCs in the negative controls when using the headspace technique. An added advantage of headspace vials is that they can be used in an auto sampler, so that GC/MS analysis can be run for multiple samples, decreasing variability between samples.

To optimize our headspace approach, we considered the effect of several experimental parameters. Screening of different SPME fiber compositions revealed that the PDMS/DVB/Carboxen polymer provided good sensitivity for alcohols, aldehydes, ketones, and carboxylic acids. Likewise, we found that a 40 min SPME incubation time allowed for SPME-GC/MS analysis per sample of <1 h, and retained good VOC extraction efficiency. A time course of *P. aeruginosa* PAO1 growth at 30°C showed a different VOC profile compared to growth at 37°C. In particular, although 1-undecene dominated VOCs regardless of temperature, at 37°C long-chain methyl ketones such as 2-undecanone were observed in significantly higher concentrations than at 30°C. Time course data also showed that there is a buildup of VOCs within the headspace of the vial. Experimentation with different media illustrated that growth substrates can alter VOC profiles. Notably, however, even growth on minimal media produced measurable quantities of VOCs from *P. aeruginosa*. The use of minimal media was advantageous in that it reduced VOC contaminants; for example, quantities of benzaldehyde emitted from MOPS minimal media were far lower compared to tryptic soy agar. Understanding and minimizing background VOCs can be beneficial for compound identification, particularly for studies where the goal is to explore the effects of microbial VOCs on species interactions.

A key benefit of our VOC analysis workflow is the streamlining that it provides. Our approach allows for higher throughput analysis of microbial VOCs. Accordingly, we demonstrated the benefits of our headspace approach by screening eight bacterial strains for VOC production on three media formulations. This experiment would have been impractical using a manual SPME holder requiring 4 h incubation times. Further, standard VOC workflows would have yielded complicated data with decreased signal to noise ratios, impeding interpretation of results. Our cross-strain comparison provides insight into the VOC profiles of different bacterial strains under a variety of growth conditions. *P. aeruginosa* was found to produce long chain alcohols and alkenes, as well as some short-chain ketones. 1-undecene biosynthesis in *Pseudomonas* has been identified as originating from undecanoic acid through the mechanism of a non-heme iron oxygenase, UndA (Rui et al., 2014). We observe, in addition to 1-undecene, 2-undecanol and 2-undecanone. Strains of *S. epidermidis* were found to produce significant amounts of long-chain methyl ketones, but fewer long-chain alcohols, aldehydes, and alkenes. This result is attributed to different metabolic activity for fatty acid biosynthesis products when compared to *P. aeruginosa*. For other species of *Staphylococcus*, the biosynthesis of long-chain methyl ketones has been recognized as incomplete β-oxidation of fatty acids (Engelvin et al., 2000; Fadda et al., 2002).

The other bacterial strains studied did not produce large quantities of VOCs as detected by our SPME-GC/MS method; however, it is interesting to note that *B. epidermidis* and *C. xerosis* were found to produce 3-methyl-1-butanol, where the first *S. epidermidis* strain produced 3-methylbutanoic acid. Potentially these bacteria are converting 3-methylbutanal that exists in the media, but in differing ways, where the former species are reducing the aldehyde, but the latter strain is oxidizing 3-methyl-1-butanol to 3-methylbutanoic acid. It is also interesting that different strains of *S. epidermidis* produce different VOCs. For example, the third strain of *S. epidermidis* produces 3-methylbutanal, whereas the second strain produces neither. These results highlight the importance of rapid culture sampling, as even variations between strains of the same bacterial species can cause measureable differences in produced VOCs.

## Conclusion

The ability of SPME-GCMS to analyze bacterial culture directly grown in headspace vials quickly provides a high throughput screening strategy for the study of microbial produced VOCs. This work will support the characterization of microbial diversity in VOC production, as well as enable the development of systems with defined VOC profiles. Due to the growing appreciation for the role of microbiomes in health, agriculture, and the environment, we believe that such capabilities for efficiently quantifying many different taxa will be increasingly important.

## Author contributions

CT: Designed and executed growth experiments and analysis; EL: Designed and executed GCMS analysis of growth experiments; AE: Designed and executed GCMS method development; RM: Designed and advised GCMS development; DK: Designed and advised growth and GCMS analysis.

### Conflict of interest statement

The authors declare that the research was conducted in the absence of any commercial or financial relationships that could be construed as a potential conflict of interest.

## Supplementary material

Figure S1 Chemical standards compared to volatiles from *P. aeruginosa* PAO1. **(A)** Chromatograms from chemical standards were collected independently then truncated and overlaid to show retention time. 1-undecene curve is gray to distinguish from 2-nonanone curve given similar retention times. **(B)** 30°C incubation, **(C)** 37°C incubation. Shapes are filled when confirmed by co-elution with chemical standards, hollow when identified only through library matching. The compounds are labeled as follows: • = 1-nonene; ◦ = 2-heptanone; □ = 2-heptanol; ▴ = 1-undecene; ■ = 2-nonanone; ♦ = 2-undecanone; ▾ = 2-undecanol; ♢ = 2-tridecanone. Peaks labeled as X are identified as siloxane contaminants.

Table S1 Custom library for putative compound identification.

## References

[B1] Amann A. Costelloc Bde L. Miekisch W. Schubert J. Buszewski B. Pleil J.. (2014). The human volatilome: volatile organic compounds (VOCs) in exhaled breath, skin emanations, urine, feces and saliva. J. Breath Res. 8:34001. 10.1088/1752-7155/8/3/034001 24946087

[B2] Arrieta M. C. Stiemsma L. T. Dimitriu P. A. Thorson L. Russell S. Yurist-Doutsch S.. (2015). Early infancy microbial and metabolic alterations affect risk of childhood asthma. Sci. Transl. Med. 7, 307ra152. 10.1126/scitranslmed.aab2271 26424567

[B3] Asensio D. Pe-uelas J. Filella I. Llusià J. (2007). On-line screening of soil VOCs exchange responses to moisture, temperature and root presence. Plant Soil 291, 249–261. 10.1007/s11104-006-9190-4

[B4] Bernier U. R. Kline D. L. Schreck C. E. Yost R. A. Barnard D. R. (2002). Chemical analysis of human skin emanations: comparison of volatiles from humans that differ in attraction of *Aedes aegypti* (Diptera: Culicidae). J. Am. Mosq. Control Assoc. 18, 186–195. Available online at: https://www.biodiversitylibrary.org/content/part/JAMCA/JAMCA_V18_N3_P186-195.pdf 12322940

[B5] Bernier U. R. Kline D. L. Barnard D. R. Schreck C. E. Yost R. A. (2000). Analysis of human skin emanations by gas chromatography/mass spectrometry. 2. Identification of volatile compounds that are candidate attractants for the yellow fever mosquito (*Aedes aegypti*). Anal. Chem. 72, 747–756. 10.1021/ac990963k 10701259

[B6] Blasioli S. Biondi E. Samudrala D. Spinelli F. Cellini A. Bertaccini A.. (2014). Identification of volatile markers in potato brown rot and ring rot by combined GC-MS and PTR-MS techniques: study on *in vitro* and *in vivo* samples. J. Agric. Food Chem. 62, 337–347. 10.1021/jf403436t 24313381

[B7] Bos L. D. Sterk P. J. Schultz M. J. (2013). Volatile metabolites of pathogens: a systematic review. PLOS Pathog. 9:e1003311. 10.1371/journal.ppat.1003311 23675295 PMC3649982

[B8] Braks M. A. Anderson R. A. Knols B. G. (1999). Infochemicals in mosquito host selection: human skin microflora and plasmodium parasites. Parasitol. Today 15, 409–413. 10.1016/S0169-4758(99)01514-8 10481153

[B9] Brun R. Blum J. Chappuis F. Burri C. (2017). Human African trypanosomiasis. Lancet 375, 148–159. 10.1016/S0140-6736(09)60829-1 19833383

[B10] Bunge M. Araghipour N. Mikoviny T. Dunkl J. Schnitzhofer R. Hansel A.. (2008). On-line monitoring of microbial volatile metabolites by proton transfer reaction-mass spectrometry. Appl. Environ. Microbiol 74, 2179–2186. 10.1128/AEM.02069-07 18245241 PMC2292582

[B11] Capozzi V. Makhoul S. Aprea E. Romano A. Cappellin L. Sanchez Jimena A.. (2016). PTR-MS characterization of VOCs associated with commercial aromatic bakery yeasts of wine and beer origin. Molecules 21:483. 10.3390/molecules21040483 27077836 PMC6274548

[B12] Chen H. Fujita M. Feng Q. Clardy J. Fink G. R. (2004). Tyrosol is a quorum-sensing molecule in Candida albicans. Proc. Natl. Acad. Sci. U.S.A. 101, 5048–5052. 10.1073/pnas.0401416101 15051880 PMC387371

[B13] Cowman A. F. Healer J. Marapana D. Marsh K. (2016). Malaria: biology and disease. Cell 167, 610–624. 10.1016/j.cell.2016.07.055 27768886

[B14] Crespo E. Cristescu S. M. de Ronde H. Kuijper S. Kolk A. H. Anthony R. M.. (2011). Proton transfer reaction mass spectrometry detects rapid changes in volatile metabolite emission by *Mycobacterium smegmatis* after the addition of specific antimicrobial agents. J. Microbiol. Methods 86, 8–15. 10.1016/j.mimet.2011.01.025 21277343

[B15] Critchley A. Elliott T. S. Harrison G. Mayhew C. A. Thompson J. M. Worthington T. (2004). The proton transfer reaction mass spectrometer and its use in medical science: applications to drug assays and the monitoring of bacteria. Int. J. Mass Spectrom. 239, 235–241. 10.1016/j.ijms.2004.08.008

[B16] Enderby B. Smith D. Carroll W. Lenney W. (2009). Hydrogen cyanide as a biomarker for *Pseudomonas aeruginosa* in the breath of children with cystic fibrosis. Pediatr. Pulmonol. 44, 142–147. 10.1002/ppul.20963 19148935

[B17] Engelvin G. Feron G. Perrin C. Mollé D. Talon R. (2000). Identification of β-oxidation and thioesterase activities in Staphylococcus carnosus 833 strain. FEMS Microbiol. Lett. 190, 115–120. 10.1016/S0378-1097(00)00302-5 10981700

[B18] Ergönül O. (2006). Crimean-Congo haemorrhagic fever. Lancet Infect. Dis. 6, 203–214. 10.1016/S1473-3099(06)70435-2 16554245 PMC7185836

[B19] Fadda S. Lebert A. Leroy-Sétrin S. Talon R. (2002). Decarboxylase activity involved in methyl ketone production by *Staphylococcus carnosus* 833, a strain used in sausage fermentation. FEMS Microbiol. Lett. 210, 209–214. 10.1111/j.1574-6968.2002.tb11182.x 12044676

[B20] Filipiak W. Sponring A. Baur M. M. Filipiak A. Ager C. Wiesenhofer H.. (2012). Molecular analysis of volatile metabolites released specifically by *Staphylococcus aureus* and pseudomonas aeruginosa. BMC Microbiol. 12:113. 10.1186/1471-2180-12-113 22716902 PMC3444334

[B21] Gallagher M. Wysocki C. J. Leyden J. J. Spielman A. I. Sun X. Preti G. (2008). Analyses of volatile organic compounds from human skin. Br. J. Dermatol. 159, 780–791. 10.1111/j.1365-2133.2008.08748.x 18637798 PMC2574753

[B22] Gilchrist F. Simms H. Alcock A. Jones A. Smith D. Spanel P. (2012b). Is hydrogen cyanide a marker of burkholderia cepacia complex infection? Thorax 67:A102 10.1136/thoraxjnl-2012-202678.330 PMC3889779 23966502

[B23] Gilchrist F. J. Alcock A. Belcher J. Brady M. Jones A. Smith D.. (2011). Variation in hydrogen cyanide production between different strains of *Pseudomonas aeruginosa*. Eur. Respir. J. 38, 409–414. 10.1183/09031936.00166510 21273393

[B24] Gilchrist F. J. Sims H. Alcock A. Belcher J. Jones A. M. Smith D. (2012a). Quantification of hydrogen cyanide and 2-aminoacetophenone in the headspace of *Pseudomonas aeruginosa* cultured under biofilm and planktonic conditions. Anal. Methods 4, 3661–3665. 10.1039/c2ay25652e

[B25] Gray C. M. Monson R. K. Fierer N. (2010). Emissions of volatile organic compounds during the decomposition of plant litter. J. Geophys. Res. Biogeosciences 115:G03015 10.1029/2010JG001291

[B26] Hammack L. Bromel M. Duh F. M. Gassner G. (1987). Reproductive factors affecting responses of the screwworm fly, *Cochliomyia hominivorax* (Diptera: Calliphoridae), to an attractant of bacterial origin. Ann. Entomol. Soc. Am. 80, 775–780. 10.1093/aesa/80.6.775

[B27] Haze S. Gozu Y. Nakamura S. Kohno Y. Sawano K. Ohta H.. (2001). 2-Nonenal newly found in human body odor tends to increase with aging. J. Invest. Dermatol. 116, 520–524. 10.1046/j.0022-202x.2001.01287.x 11286617

[B28] Hogan D. A. (2006). Quorum sensing: alcohols in a social situation. Curr. Biol. 16, R457–R458. 10.1016/j.cub.2006.05.035 16782000

[B29] Holm E. S. Adamsen A. P. Feilberg A. Schäfer A. Løkke M. M. Petersen M. A. (2013). Quality changes during storage of cooked and sliced meat products measured with PTR-MS and HS-GC–MS. Meat Sci. 95, 302–310. 10.1016/j.meatsci.2013.04.046 23747622

[B30] Jiang R. Cudjoe E. Bojko B. Abaffy T. Pawliszyn J. (2013). A non-invasive method for *in vivo* skin volatile compounds sampling. Anal. Chim. Acta 804, 111–119. 10.1016/j.aca.2013.09.056 24267071

[B31] Kameyama S. Tanimoto H. Inomata S. Suzuki K. Komatsu D. D. Hirota A. (2011). Application of PTR-MS to an incubation experiment of the marine diatom *Thalassiosira pseudonana*. Geochem. J. 45, 355–363. 10.2343/geochemj.1.0127

[B32] Kennedy P. G. (2013). Clinical features, diagnosis, and treatment of human African trypanosomiasis (sleeping sickness). Lancet Neurol. 12, 186–194. 10.1016/S1474-4422(12)70296-X 23260189

[B33] Labows J. N. McGinley K. J. Webster G. F. Leyden J. J. (1980). Headspace analysis of volatile metabolites of *Pseudomonas aeruginosa* and related species by gas chromatography- mass spectrometry. J. Clin. Microbiol. 12, 521–526. 6775012 10.1128/jcm.12.4.521-526.1980 PMC273628

[B34] Lazazzara V. Perazzolli M. Pertot I. Biasioli F. Puopolo G. Cappellin L. (2017). Growth media affect the volatilome and antimicrobial activity against *Phytophthora infestans* in four Lysobacter type strains. Microbiol. Res. 201, 52–62. 10.1016/j.micres.2017.04.015 28602402

[B35] Machiels K. Joossens M. Sabino J. De Preter V. Arijs I. Eeckhaut V.. (2014). A decrease of the butyrate-producing species *Roseburia hominis* and *Faecalibacterium prausnitzii* defines dysbiosis in patients with ulcerative colitis. Gut 63, 1275–1283. 10.1136/gutjnl-2013-304833 24021287

[B36] Makhoul S. Romano A. Capozzi V. Spano G. Aprea E. Cappellin L. (2015). Volatile compound production during the bread-making process: effect of flour, yeast and their interaction. Food Bioprocess. Technol. 8, 1925–1937. 10.1007/s11947-015-1549-1

[B37] Mallette N. D. Knighton W. B. Strobel G. A. Carlson R. P. Peyton B. M. (2012). Resolution of volatile fuel compound profiles from *Ascocoryne sarcoides*: a comparison by proton transfer reaction-mass spectrometry and solid phase microextraction gas chromatography-mass spectrometry. AMB Express 2:23. 10.1186/2191-0855-2-23 22480438 PMC3402149

[B38] Mboera L. E. G. Knols B. G. J. Takken W. Torre A. della (1997). The response of *Anopheles gambiae* s.l. and *A. funestus* (Diptera: Culicidae) to tents baited with human odour or carbon dioxide in Tanzania. Bull. Entomol. Res. 87, 173–178. 10.1017/S0007485300027322

[B39] O'Hara M. Mayhew C. (2009). A preliminary comparison of volatile organic compounds in the headspace of cultures of *Staphylococcus aureus* grown in nutrient, dextrose and brain heart bovine broths measured using a proton transfer reaction mass spectrometer. J. Breath Res. 3:027001. 10.1088/1752-7155/3/2/027001 21383456

[B40] Pavlou A. K. Magan N. Sharp D. Brown J. Barr H. Turner A. P. (2000). An intelligent rapid odour recognition model in discrimination of *Helicobacter pylori* and other gastroesophageal isolates *in vitro*. Biosens. Bioelectron. 15, 333–342. 10.1016/S0956-5663(99)00035-4 11219746

[B41] Petnicki-Ocwieja T. Brissette C. A. (2015). Lyme disease: recent advances and perspectives. Front. Cell. Infect. Microbiol. 5:27. 10.3389/fcimb.2015.00027 25883907 PMC4381694

[B42] Pichersky E. Gershenzon J. (2002). The formation and function of plant volatiles: perfumes for pollinator attraction and defense. Curr. Opin. Plant Biol. 5, 237–243. 10.1016/S1369-5266(02)00251-0 11960742

[B43] Ramirez K. S. Lauber C. L. Fierer N. (2010). Microbial consumption and production of volatile organic compounds at the soil-litter interface. Biogeochemistry 99, 97–107. 10.1007/s10533-009-9393-x

[B44] Riazanskaia S. Blackburn G. Harker M. Taylor D. Thomas C. L. P. (2008). The analytical utility of thermally desorbed polydimethylsilicone membranes for *in-vivo* sampling of volatile organic compounds in and on human skin. Analyst 133, 1020–1027. 10.1039/b802515k 18645643

[B45] Ríos-Covián D. Ruas-Madiedo P. Margolles A. Gueimonde M. de los Reyes-Gavilán C. G. Salazar N. (2016). Intestinal short chain fatty acids and their link with diet and human health. Front. Microbiol. 7:185. 10.3389/fmicb.2016.00185 26925050 PMC4756104

[B46] Romano A. Capozzi V. Spano G. Biasioli F. (2015). Proton transfer reaction–mass spectrometry: online and rapid determination of volatile organic compounds of microbial origin. Appl. Microbiol. Biotechnol. 99, 3787–3795. 10.1007/s00253-015-6528-y 25808516

[B47] Rui Z. Li X. Zhu X. Liu J. Domigan B. Barr I.. (2014). Microbial biosynthesis of medium-chain 1-alkenes by a nonheme iron oxidase. Proc. Natl. Acad. Sci. U.S.A. 111, 18237–18242. 10.1073/pnas.1419701112 25489112 PMC4280606

[B48] Rybakova D. Rack-Wetzlinger U. Cernava T. Schaefer A. Schmuck M. Berg G. (2017). Aerial warfare: a volatile dialogue between the plant pathogen *Verticillium longisporum* and its antagonist *Paenibacillus polymyxa*. Front. Plant Sci. 8:1294. 10.3389/fpls.2017.01294 28798756 PMC5529406

[B49] Ryu C. M. Farag M. A. Hu C. H. Reddy M. S. Wei H. X. Pare P. W.. (2003). Bacterial volatiles promote growth in Arabidopsis. Proc. Natl. Acad. Sci. U.S.A. 100, 4927–4932. 10.1073/pnas.0730845100 12684534 PMC153657

[B50] Savelev S. Perry J. Bourke S. Taylor R. Fisher A. Petrie M. (2010). Identification of *Pseudomonas aeruginosa* infection via volatile organic compounds in sputum headspace gases. Thorax 65, A12–A13. 10.1136/thx.2010.150912.21

[B51] Scott-Thomas A. J. Syhre S. Pattemore P. K. Epton M. Laing R. Pearson J.. (2010). 2-Aminoacetophenone as a potential breath biomarker for *Pseudomonas aeruginosa* in the cystic fibrosis lung. BMC Pulm. Med. 10:56. 10.1186/1471-2466-10-56 21054900 PMC2989937

[B52] Simmons C. P. Farrar J. J. van Vinh Chau N. Wills B. (2012). Dengue. N. Engl. J. Med. 366, 1423–1432. 10.1056/NEJMra1110265 22494122

[B53] Tait E. Perry J. D. Stanforth S. P. Dean J. R. (2014). Identification of volatile organic compounds produced by bacteria using HS-SPME-GC–MS. J. Chromatogr. Sci. 52, 363–373. 10.1093/chromsci/bmt042 23661670

[B54] Trowell S. Berna A. Padovan B. Locke V. (2015). Method of Detecting Plasmodium Infection. Available online at: https://www.google.com/patents/WO2015077843A1?cl=en

[B55] Utama I. M. Wills R. B. Ben-yehoshua S. Kuek C. (2002). *in vitro* efficacy of plant volatiles for inhibiting the growth of fruit and vegetable decay microorganisms. J. Agric. Food Chem. 50, 6371–6377. 10.1021/jf020484d 12381119

[B56] Verhulst N. O. Andriessen R. Groenhagen U. Bukovinszkiné Kiss G. Schulz S. Takken W.. (2011a). Differential attraction of malaria mosquitoes to volatile blends produced by human skin bacteria. PLoS ONE 5:e15829. 10.1371/journal.pone.0015829 21209854 PMC3012726

[B57] Verhulst N. O. Beijleveld H. Qiu Y. T. Maliepaard C. Verduyn W. Haasnoot G. W.. (2013). Relation between HLA genes, human skin volatiles and attractiveness of humans to malaria mosquitoes. Infect. Genet. Evol. 18, 87–93. 10.1016/j.meegid.2013.05.009 23688850

[B58] Verhulst N. O. Qiu Y. T. Beijleveld H. Maliepaard C. Knights D. Schulz S.. (2011b). Composition of human skin microbiota affects attractiveness to malaria mosquitoes. PLoS ONE 6:e28991. 10.1371/journal.pone.0028991 22216154 PMC3247224

[B59] Weise T. Kai M. Gummesson A. Troeger A. von Reuss S. Piepenborn S.. (2012). Volatile organic compounds produced by the phytophatogenic bacterium *Xanthomonas campestris* pv. vesicatoria 85-10. Beilstein J. Org. Chem. 8, 579–596. 10.3762/bjoc.8.65 22563356 PMC3343284

[B60] Wu Q. L. Guo W. Q. Zheng H. S. Luo H. C. Feng X. C. Yin R. L.. (2016). Enhancement of volatile fatty acid production by co-fermentation of food waste and excess sludge without pH control: the mechanism and microbial community analyses. Bioresour. Technol. 216, 653–660. 10.1016/j.biortech.2016.06.006 27289056

[B61] Zechman J. M. Labows J. N. Jr. (1985). Volatiles of *Pseudomonas aeruginosa* and related species by automated headspace concentration – gas chromatography. Can. J. Microbiol. 31, 232–237. 10.1139/m85-045 3924382

