# Supplementary figures and images for: Direct Growth of Bacteria in Headspace Vials Allows for Screening of Volatiles by Gas Chromatography Mass Spectrometry
Source: Front Microbiol. 2018 Mar 20;9:491. doi: 10.3389/fmicb.2018.00491 (PMC5890184; doi:10.3389/fmicb.2018.00491)

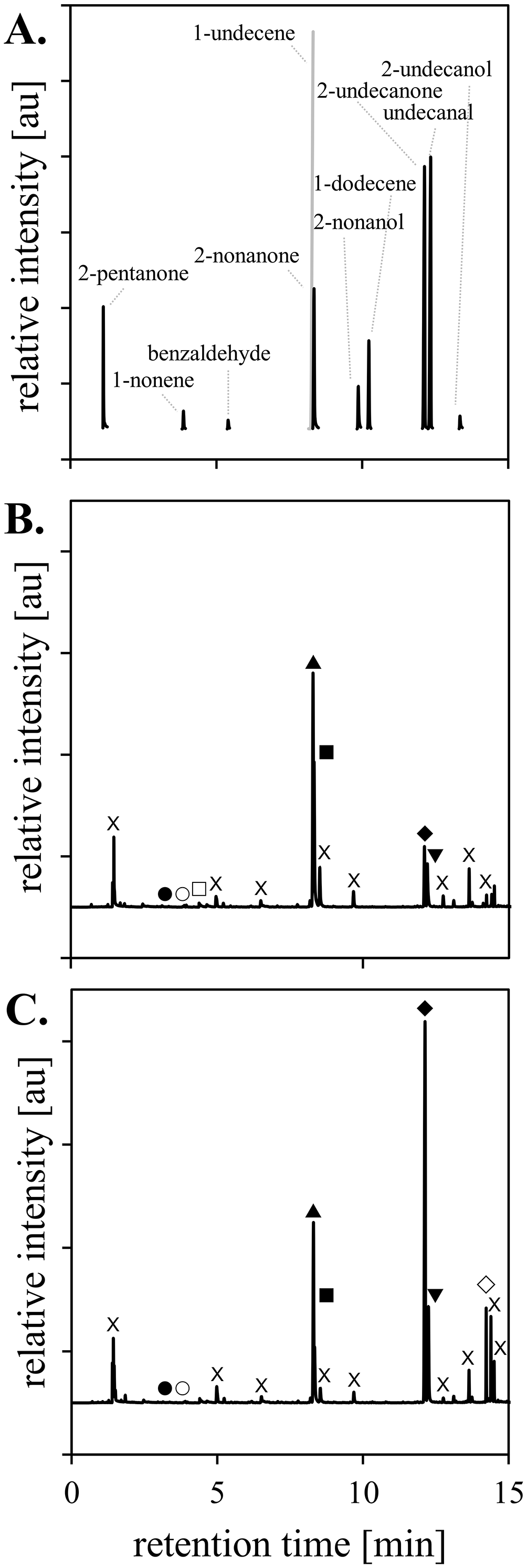

Supplement: Figure S1 — Chemical standards compared to volatiles from P. aeruginosa PAO1. (A) Chromatograms from chemical standards were collected independently then truncated and overlaid to show retention time. 1-undecene curve is gray to distinguish from 2-nonanone curve given similar retention times. (B) 30°C incubation, (C) 37°C incubation. Shapes are filled when confirmed by co-elution with chemical standards, hollow when identified only through library matching. The compounds are labeled as follows: • = 1-nonene; ◦ = 2-heptanone; □ = 2-heptanol; ▴ = 1-undecene; ■ = 2-nonanone; ♦ = 2-undecanone; ▾ = 2-undecanol; ♢ = 2-tridecanone. Peaks labeled as X are identified as siloxane contaminants. [file Image1.TIF]
